# Association of a standardized nursing protocol with postoperative complications following open radical gastrectomy: a retrospective cohort study

**DOI:** 10.1515/med-2026-1460

**Published:** 2026-06-01

**Authors:** Na Yan, Qing Hong, Jinghui Yuan, Wei Fei

**Affiliations:** The Affiliated Suqian Hospital of Xuzhou Medical University, Suqian, Jiangsu, P. R. China

**Keywords:** gastric cancer, radical gastrectomy, operating room nursing, standardized protocol, postoperative complications

## Abstract

**Objectives:**

To evaluate the independent association of a standardized, multi-modal intraoperative nursing protocol on postoperative complications in patients undergoing open radical gastrectomy for gastric cancer.

**Methods:**

This retrospective cohort study included 89 patients who underwent open radical gastrectomy. Patients were categorized into a standardized care group (n=44) receiving a multi-modal nursing protocol and a non-standardized care group (n=45). The primary outcome was 30-day complications (Clavien–Dindo ≥II). Multivariable logistic regression adjusted for confounders.

**Results:**

A total of 89 patients were included (standardized care group, n=44; non-standardized care group, n=45). The total complication rate was significantly lower in the Standardized Care Group (22.7 % vs. 48.9 %, p<0.01). After adjustment, standardized nursing care was independently associated with reduced complications (adjusted OR=0.32; 95 % CI: 0.12–0.85; p=0.022). Intraoperative hypothermia (OR=3.45; 95 % CI: 1.42–8.38) and operative time ≥200 min (OR=2.89; 95 % CI: 1.18–7.07) were identified as independent risk factors.

**Conclusions:**

In the context of open gastrectomy, a systematic intraoperative nursing protocol is significantly and independently associated with reduced postoperative morbidity, primarily by being associated with lower rates of procedure-specific risks such as hypothermia and prolonged operative time. This study provides quantitative evidence to support reconceptualizing structured operating room nursing from a supportive role to a core component of therapeutic intervention associated with improved surgical outcomes.

## Introduction

Gastric cancer remains a leading cause of cancer-related mortality worldwide. Postoperative complications remain a major challenge following open radical gastrectomy for gastric cancer [1], impairing recovery and survival. While surgical technique and patient factors are well-studied, modifiable intraoperative risks–such as hypothermia and prolonged operative time–are often overlooked in outcome models [2], 3].

The operating room nurse’s role has traditionally been viewed as supportive. However, evidence suggests that targeted nursing interventions can directly influence these key risk factors [4], 5]. In open surgery, where physiological trespass is significant, structured nursing care may therefore shift from a supportive to a therapeutic function. Despite growing recognition of the importance of intraoperative care, whether structured nursing interventions independently improve surgical outcomes remains unquantified.

The standardized intraoperative nursing protocol evaluated in this study comprises four evidence-based components: active temperature management, goal-directed fluid balance monitoring, phase-based efficient instrument management, and enhanced compliance with infection prevention bundles. Although each of these elements is individually recommended in perioperative guidelines, they have not been systematically integrated into a nurse-led protocol specifically for patients undergoing open radical gastrectomy. Furthermore, whether such a structured nursing intervention independently improves surgical outcomes in this particular high-risk population remains unquantified. This study was therefore designed to address this gap. Therefore, we hypothesized that implementation of a standardized intraoperative nursing protocol is an independent protective factor against postoperative complications in patients undergoing open radical gastrectomy. The primary research question was: Does a systematic, nurse-led intraoperative protocol reduce the risk of major complications independently of patient and tumor characteristics? [6].

## Materials and methods

### Study design and participants

This single-center retrospective cohort study consecutively enrolled adult patients (≥18 years) who underwent elective open radical gastrectomy (including total, distal, proximal, and multivisceral resections) for gastric cancer at our hospital between January 1, 2020, and December 31, 2024. All operations and nursing care were completed by the same surgical and nursing team. Exclusion criteria were: (1) emergency surgery; (2) palliative or exploratory surgery without resection; (3) laparoscopic or robot-assisted procedures; (4) active infection within 30 days preoperatively. A total of 11 patients were excluded due to missing data on key variables essential for the primary analysis: operative time (n=4), intraoperative temperature recordings (n=5), and postoperative pain scores (n=2). These missing data occurred predominantly in the earlier part of the study period (before protocol implementation), when routine documentation of these variables was not yet standardized. After exclusions, 89 patients were included in the final analysis.

### Group definition and intervention

To assess the association of the systematic nursing protocol, a historical cohort design was employed, utilizing the date of hospital-wide protocol implementation as the objective cutoff. All patients meeting the inclusion criteria within the study period (January 2020–December 2024) were assigned to one of two cohorts based on their surgery date:

Non-standardized care group (control cohort): Comprised patients who underwent surgery before the mandatory implementation date (July 1, 2022). During this period, intraoperative care was delivered according to the routine, non-protocolized practices prevailing at the time. These practices included intermittent temperature monitoring without mandatory active warming, fluid administration based on clinical judgment without a structured goal-directed protocol, and non-standardized instrument table organization. No formal infection prevention bundles were enforced.

Standardized care group (intervention cohort): Comprised patients who underwent surgery on or after the mandatory implementation date (July 1, 2022). For these patients, the “Standardized Nursing Protocol for Radical Gastrectomy” was applied as the standard of care.

This protocol, developed in accordance with the Joanna Briggs Institute model of evidence-based healthcare and informed by prior institutional practice [7], centered on four structured modules: ① Active Temperature Management (pre-warming before induction, mandatory use of forced-air warming blankets, warmed fluid infusion) [8]; ② Goal-directed fluid balance monitoring [9] (hourly recording and communication of intake/output by nurses, collaborative adjustment with anesthesiologists) [10]; ③ Phase-Based Efficient Instrument Management (standardized instrument table setup and passing sequences); ④ Enhanced Compliance with Infection Prevention Bundles (timing verification of antibiotic administration, maintenance of sterile barrier integrity, standardized management of intraoperative irrigation fluids).

### Data collection and variable definitions

Operative time was dichotomized at ≥200 min based on clinically meaningful thresholds established in previous large-cohort studies of open gastrectomy [11]. This cutoff was defined prior to statistical analysis. Two trained research nurses independently collected data using a standardized extraction form via the electronic medical record and anesthesia information systems.

Variables collected included:

Demographics and baseline characteristics: Age, sex, body mass index (BMI), American Society of Anesthesiologists (ASA) physical status classification. Baseline variables were collected from the preoperative assessment records within 30 days prior to surgery.

Surgical variables: Surgical procedure, operative time, estimated blood loss, combined organ resection.

Pathological variables: Tumor location, pT stage (American Joint Committee on Cancer AJCC 7th edition), histological grade, lymph node ratio (LNR).

Key intraoperative nursing process indicators: Occurrence of intraoperative hypothermia (core temperature <36.0 °C), total intraoperative fluid volume, documentation of fluid balance monitoring sheet.

Outcome measures: The primary outcome was the incidence of total postoperative complications within 30 days (Clavien–Dindo classification≥Grade II) [12]. Secondary outcomes included rates of specific complications (e.g., surgical site infection, pneumonia, anastomotic leak, intra-abdominal infection) and postoperative length of hospital stay (LOS).

### Statistical analysis

SPSS version 26.0 was used. Continuous variables were presented as mean ± standard deviation or median (interquartile range, IQR) based on normality tests (Shapiro–Wilk), compared using independent t-tests or Mann–Whitney U tests. Categorical variables were presented as counts (%), compared using Chi-square or Fisher’s exact tests.

To evaluate the independent association of standardized nursing care on complications, univariate analysis was first performed to screen potential confounders (p<0.10 for inclusion). Subsequently, a multivariate binary logistic regression model was constructed. The main independent variable was “receipt of standardized care.” Variables identified in univariate analysis (p<0.10) and clinically relevant variables (e.g., age, surgical procedure, pT stage) were included in the model to calculate adjusted odds ratios (ORs) with 95 % confidence intervals (CIs). All tests were two-sided, with p<0.05 considered statistically significant.

### Ethics and consent

This study was conducted in accordance with the Declaration of Helsinki and approved by the Institutional Review Board of the Affiliated Suqian Hospital of Xuzhou Medical University (Approval no. IRB-S-2020-012). Due to the retrospective nature of the study and the use of anonymized clinical data, the requirement for informed consent was waived.

## Results

### Patient baseline characteristics and intraoperative process measures

A total of 89 patients were included in the final analysis: 44 in the standardized care group and 45 in the non-standardized care group. The two groups were well-balanced regarding baseline demographic and clinicopathological characteristics, including age, sex, BMI, ASA classification, pathological T and N stage, and type of gastrectomy performed (all p>0.05). The standardized care group had a significantly shorter median operative time (185 vs. 210 min, p=0.018). There was a non-significant trend towards lower estimated blood loss in the standardized care group (Table 1).

**Table 1:** Baseline characteristics of the study populationen.

Characteristic	Standardized care (n=44)	Non-standardized care (n=45)	p-Value
**Demographics**			
Age, years, mean ± SD	60.5 ± 10.1	61.9 ± 10.9	0.541
Male sex, n (%)	28 (63.6)	31 (68.9)	0.604
BMI, kg/m^2^, mean ± SD	22.1 ± 3.0	21.8 ± 2.8	0.635
ASA classification≥III, n (%)	11 (25.0)	14 (31.1)	0.524
**Tumor and surgical factors**			
pT stage≥T3, n (%)	32 (72.7)	35 (77.8)	0.582
pN stage≥N1, n (%)	36 (81.8)	38 (84.4)	0.741
Type of resection (total gastrectomy), n (%)	20 (45.5)	21 (46.7)	0.910
Operative time, min, median [IQR]	185 [160–215]	210 [180–250]	0.018
Estimated blood loss, mL, median [IQR]	200 [150–300]	250 [180–350]	0.089

Regarding intraoperative process measures, the standardized care group demonstrated significantly better performance: the proportion of patients with intraoperative hypothermia was 9.1 % vs. 31.1 % (p=0.008), and fluid balance monitoring was documented in 100 % vs. 40.0 % (p<0.001) (Table 2).

**Table 2:** Intraoperative process measures by group.

Measure	Standardized care (n=44)	Non-standardized care (n=45)	p-Value
Intraoperative hypothermia (<36.0 °C), n (%)	4 (9.1)	14 (31.1)	0.008
Fluid balance monitoring documented, n (%)	44 (100)	18 (40.0)	<0.001

Univariate analysis identified several factors associated with postoperative complications (Table 3). Patients who developed complications had higher proportions of pT stage≥T3 (93.1 % vs. 70.2 %, p=0.020), pN stage≥N1 (100 % vs. 78.9 %, p=0.008), operative time ≥200 min (69.0 % vs. 31.6 %, p=0.001), and intraoperative hypothermia (34.5 % vs. 14.0 %, p=0.028). Estimated blood loss ≥300 mL showed a non-significant trend towards association with complications (41.4 % vs. 22.8 %, p=0.071). These variables were entered into the multivariate logistic regression model.

**Table 3:** Univariate analysis of factors associated with postoperative complications.

Variable	No complication (n=57)	Complication (n=29)	OR (95 % CI)	p-Value
Age ≥70 years, n (%)	16 (28.1)	12 (41.4)	1.81 (0.71–4.62)	0.211
Male sex, n (%)	35 (61.4)	16 (55.2)	0.77 (0.31–1.92)	0.604
ASA≥III, n (%)	14 (24.6)	11 (37.9)	1.87 (0.71–4.91)	0.204
pT stage≥T3, n (%)	40 (70.2)	27 (93.1)	5.74 (1.24–26.56)	0.020
pN stage≥N1, n (%)	45 (78.9)	29 (100)	–	0.008
Total gastrectomy, n (%)	22 (38.6)	14 (48.3)	1.48 (0.60–3.66)	0.391
Operative time ≥200 min, n (%)	18 (31.6)	20 (69.0)	4.82 (1.82–12.78)	0.001
EBL ≥300 mL, n (%)	13 (22.8)	12 (41.4)	2.38 (0.91–6.23)	0.071
Intraoperative hypothermia, n (%)	8 (14.0)	10 (34.5)	3.24 (1.11–9.48)	0.028
Fluid balance documented, n (%)	42 (73.7)	20 (69.0)	0.79 (0.29–2.15)	0.643

pN stage could not be estimated due to zero events in the no-complication group.

### Postoperative outcomes

The standardized care group experienced a significantly lower overall rate of postoperative complications (22.7 % vs. 48.9 %, p=0.010). A trend towards a shorter median postoperative length of stay was observed in the standardized care group, although the difference did not reach statistical significance (12 vs. 14 days, p=0.057). Rates of specific complications, such as surgical site infection and pneumonia, were lower in the intervention group, though these differences did not reach statistical significance (Table 4).

**Table 4:** Postoperative outcomes by group.

Outcome	Standardized care (n=44)	Non-standardized care (n=45)	p-Value
Any complication (Clavien–Dindo≥II), n (%)	10 (22.7)	22 (48.9)	0.010
Surgical site infection, n (%)	3 (6.8)	8 (17.8)	0.116
Pneumonia, n (%)	2 (4.5)	7 (15.6)	0.087
Postoperative length of stay, days, median [IQR]	12 [10–16]	14 [11–19]	0.057

### Multivariable analysis of factors associated with complications

The results of the multivariable logistic regression analysis are presented in Table 5. After adjustment for age, type of resection, and pT stage, receipt of standardized nursing care emerged as a strong independent protective factor against postoperative complications (adjusted odds ratio [OR] = 0.32; 95 % confidence interval [CI]: 0.12–0.85; p=0.022). This corresponds to a 68 % reduction in the odds of developing a complication. Intraoperative hypothermia (adjusted OR=3.45; 95 % CI: 1.42–8.38; p=0.006) and an operative time of 200 min or longer (adjusted OR=2.89; 95 % CI: 1.18–7.07; p=0.020) were identified as significant independent risk factors. Notably, the pathological T stage was not an independent predictor of complications in this model.

**Table 5:** Multivariable logistic regression analysis for factors associated with postoperative complications.

Variable	Adjusted odds ratio	95 % confidence interval	p-Value
Standardized nursing care (yes vs. no)	0.32	0.12–0.85	0.022
Intraoperative hypothermia (yes vs. no)	3.45	1.42–8.38	0.006
Operative time ≥200 min (yes vs. no)	2.89	1.18–7.07	0.020
Age ≥65 years (yes vs. no)	1.87	0.78–4.51	0.162
Type of resection (total vs. other)	1.65	0.67–4.08	0.276
pT stage (≥T3 vs. ≤T2)	1.42	0.53–3.83	0.486

## Discussion

In the present study, we found that implementation of a standardized intraoperative nursing protocol was independently associated with a 68 % reduction in the odds of postoperative complications following open radical gastrectomy (adjusted OR=0.32; 95 % CI: 0.12–0.85; p=0.022) [13], confirming our hypothesis and answering the research question in the affirmative. This finding, derived from a cohort balanced in key demographics and pathology, necessitates a fundamental reevaluation of the operating room nurse’s role in major oncologic surgery [14].

### Intercepting modifiable risks: the mechanism of protection

The protective effect may be explained, at least in part, by the mitigation of specific, nurse-sensitive risk factors–a hypothesized mechanistic pathway that warrants formal testing in future studies. Our multivariate model identified intraoperative hypothermia (OR=3.45) and prolonged operative time (OR=2.89) as significant independent drivers of morbidity. The nursing protocol functioned as a targeted countermeasure: active warming bundled with fluid management was associated with a reduction from 31.1 % to 9.1 %, while standardized instrument and workflow management likely contributed to the observed reduction in median operative time. This positions the protocol not as a collection of tasks, but as a physiological buffer system against the “second hit” of surgery, may directly intervene in the hypothesized causal pathway to complications. The shorter operative time observed in the standardized group may reflect improved workflow efficiency fostered by the protocol, which could in turn reduce exposure to surgical stress. We hypothesize that operative time may function as a mediator of the protective effect; however, formal mediation analysis was beyond the scope of this retrospective study and should be addressed in future prospective research [15].

### Interpreting the predictive value of nursing-sensitive factors

The shift in significance from pT stage in univariate analysis to nursing-sensitive factors in the multivariate model underscores the explanatory role of intraoperative care. This attenuation of pT stage’s predictive effect after adjusting for nursing-sensitive process indicators is a key finding. It suggests that, within our cohort, the prognostic information conveyed by tumor depth was, to a substantial degree, captured or mediated by modifiable intraoperative factors–factors that are directly influenced by nursing care. This does not imply that tumor biology is unimportant, but rather that its impact on short-term postoperative outcomes may be partially explainable through the quality of intraoperative physiological management.

Based on these results, we propose a broader conceptual implication: traditional risk stratification models, which predominantly rely on patient- and disease-related factors [16], may be usefully complemented by incorporating nursing-sensitive intraoperative process indicators [4], 17]. From a practice perspective, this implies a shift in nursing assessment focus–from documenting “what disease the patient has” to actively evaluating “what kind of intraoperative process we are providing.”

### Evidence for an expanded nursing role in intraoperative homeostasis management

The substantial improvement in process measures highlights the potential for protocol-driven nursing to assume a proactive role in maintaining physiological homeostasis. A key implication of these findings concerns the operating room nurse’s scope of practice. By mandating active temperature management and goal-directed fluid monitoring, the protocol effectively operationalized nurses’ responsibility for maintaining core physiological homeostasis [18]–a function traditionally viewed as ancillary to surgical and anesthetic care. Our results suggest that, when appropriately supported by institutional protocols and training, nurses can reliably perform this homeostatic governance function.

We therefore propose that the nursing role in this context be reconceptualized from passive task execution to active, protocol-driven physiological management. However, this interpretation extends beyond our direct empirical evidence and should be tested in future studies examining the relationship between protocol implementation, nurse autonomy, and patient outcomes [18].

### Toward nursing-sensitive outcome indicators: empirical grounding for quality improvement

This study identifies intraoperative hypothermia and prolonged operative time as factors strongly and independently associated with major complications after open gastrectomy. Both are measurable, modifiable, and directly influenced by nursing processes. These empirical findings provide a rationale for considering them as candidate nursing-sensitive outcome indicators–metrics that reflect the structure, process, and outcomes of nursing care.

At the practice level, these findings suggest several actionable directions. First, incorporating hypothermia rates and procedure time efficiency into routine nursing quality audits could shift the focus from compliance monitoring (e.g., “was a warming device applied?”) to outcome-oriented evaluation (e.g., “was normothermia maintained?”). Second, nursing education should strengthen pathophysiological understanding of why indicators such as hypothermia matter, transforming preventive measures from routine tasks into clinically reasoned interventions [19].

While these recommendations are grounded in our data, their implementation and impact require further investigation. Future research should prospectively evaluate whether integrating these nursing-sensitive indicators into quality benchmarking and continuing education programs leads to measurable improvements in patient outcomes.

### Limitations

This study has several limitations. First, the retrospective, single-center design and the use of a historical control group introduce potential selection bias and unmeasured confounding. Although multivariate adjustment was performed and baseline characteristics were balanced, residual confounding due to secular trends in surgical or anesthetic care cannot be fully excluded.

Second, the modest sample size (n=89) limited statistical power, and the lack of formal mediation analysis means that the hypothesized role of hypothermia and operative time as mediators remains provisional and requires confirmation in future prospective studies.

Third, the cohort consisted exclusively of patients undergoing open gastrectomy. While this homogeneity strengthens internal validity for this specific population, it limits generalizability to minimally invasive approaches.

Finally, the proposed risk-stratified nursing pathway, while grounded in our findings, has not been prospectively validated. Its clinical effectiveness, feasibility, and impact on patient outcomes warrant further investigation.

## Conclusions

This study demonstrates that standardized intraoperative nursing care is independently associated with improved surgical outcomes, reducing complication odds by 68 % after open radical gastrectomy. The protective effect was associated with lower rates of hypothermia and shorter operative time–two modifiable risk factors that may represent key mechanistic targets for nursing intervention. Based on these findings, we argue that high-quality surgical care should be redefined to include the standardization of nursing practices as a core therapeutic component, not merely an adjunct [20].
